# Transcriptome co-expression network analysis identifies key genes and regulators of ripening kiwifruit ester biosynthesis

**DOI:** 10.1186/s12870-020-2314-9

**Published:** 2020-03-06

**Authors:** Aidi Zhang, Qiuyun Zhang, Jianzhao Li, Hansheng Gong, Xinguang Fan, Yanqing Yang, Xiaofen Liu, Xueren Yin

**Affiliations:** 1 School of Food Engineering, Ludong University, Yantai, Shandong 264025 People’s Republic of China; 2 BioNanotechnology Institute, Ludong University, Yantai, Shandong 264025 People’s Republic of China; 3 College of Agriculture & Biotechnology, Zhejiang University, Zijingang Campus, Hangzhou, 310058 People’s Republic of China; 4 School of Agriculture, Ludong University, Yantai, Shandong 264025 People’s Republic of China

**Keywords:** Aroma, Kiwifruit, RNA-seq, Ester biosynthesis, Transcriptional regulation, Ripening fruit, NAC, Dof

## Abstract

**Background:**

Aroma is an important organoleptic quality for fruit and has a large influence on consumer preference. Kiwifruit esters undergo rapid and substantial changes contributing to the flavor during fruit ripening. Part of enzymes and their coding genes have been indicated potential candidates for flavor-related esters synthesis. However, there still exist obvious gaps in the biosynthetic pathways of esters and the mechanisms regulating ester biosynthesis in kiwifruit remain unknown.

**Results:**

Using gas chromatography-mass spectrometry (GC-MS), volatile compounds of kiwifruit were quantified in response to ethylene (ETH, 100 μl/l, 24 h, 20 °C) and 1-methylcyclopropene (1-MCP, 1 μl/l, 24 h, 20 °C). The results indicated that esters showed the most substantial changes enhanced by ethylene and were inhibited by 1-MCP. Correlations between RNA-seq results and concentrations of esters, constructed using Weighted Gene Co-Expression Network Analysis (WGCNA) indicated that three structural genes (fatty acid desaturase, *AdFAD1*; aldehyde dehydrogenase, *AdALDH2*; alcohol acyltransferase, *AdAT17*) had similar expression patterns that paralled the changes in total ester content, and *AdFAD1* transcripts exhibited the highest correlation. In order to search for potential regulators for ester biosynthesis, 14 previously reported ethylene-responsive transcription factors (TFs) were included in the correlation analysis with esters and their biosynthetic genes. Using dual-luciferase assay, the in vivo regulatory activities of TFs on ester biosynthetic gene promoters were investigated and the results indicated that AdNAC5 and AdDof4 (DNA binding with one finger) trans-activated and trans-suppressed the *AdFAD1* promoter.

**Conclusions:**

The present study advanced the molecular basis of ripening-related ester biosynthesis in kiwifruit by identifying three biosynthetic related genes *AdFAD1*, *AdALDH2* and *AdAT17* by transcriptome analysis, and highlighted the function of two TFs by transactivation studies.

## Background

Kiwifruit (*Actinidia deliciosa*) is one of the most recently developed and economically important fruit crops that is native to China and now has a global commercial distribution [1]. Kiwifruit are widely preferred by consumers due to their flavor and high value of nutrient compounds (eg. vitamin C) [2]. Aroma, together with sweetness and acidity generate the unique flavor of kiwifruit. The production of aroma volatile compounds is strongly ethylene-dependent [3]. In ethylene-suppressed kiwifruit, fruit softening was significantly delayed and aroma volatile production was dramatically reduced, which could be re-initiated by application of exogenous ethylene [4]. The aroma of kiwifruit is produced by a mixture of various volatile compounds, including alcohols, aldehydes, ketones, and esters [5], which makes kiwifruit a good model for understanding the biosynthesis and regulation of different volatile compounds.

Kiwifruit have long been used as material for volatile studies. The early reports on kiwifruit volatile compounds published by Young et al., identified ethyl butanoate, (E)-2-hexenal, (E)-2-hexenol, methyl and ethyl benzoate as the major components of kiwifruit aroma, by using simultaneous distillation extraction of volatiles [6]. Since then, kiwifruit volatiles have been extensively studied and expanded with 80 to 90 compounds identified so far [7, 8]. In general, aldehydes are more abundant in unripe fruit with green notes and esters with fruity notes are abundant in ripe fruit and n-hexanal, (E)-2-hexenal and ethyl butanoate have been identified as specific kiwifruit volatiles [8]. Until now, many different methods have been developed and used for the analysis of the aroma compounds in kiwifruit, such as gas chromatography-mass spectrometry (GC-MS), which have further enlarged the categories of known volatile compounds in kiwifruit [9, 10].

Esters are major aromatic components responsible for the fruity aroma, which increases drastically with kiwifruit ripening [8, 9]. The activity of lipoxygenase (LOX) enzyme contributes to this and increases markedly as fruit developed to the climacteric stage and some LOX genes are up-regulated by ethylene treatment, particularly *AdLox1* and *AdLox5* [11, 12]. Günther et al. indicated that ethylene regulated ester production in *A. chinensis* by inducing *AAT* gene expression and AAT-substrate precursor formation [13]. Based on the *Actinidia* EST database, 30 acyltransferases (ATs) were identified and phylogenetic analysis indicated 12 ATs are potential candidates for flavor-related esters synthesis [14]. However, several likely key enzymes and their coding genes involved in production of esters in kiwifruit have not been covered, such as fatty acid desaturase (FAD), hydroperoxide lyase (HPL), aldehyde dehydrogenase (ALDH). Some of the genes analyzed in kiwifruit, for instance *AdADH1* and *AdADH2* (alcohol dehydrogenase) are activated after waterlogging [15], which is not related to fruit ester production. Despite the importance of esters for kiwifruit flavor, there still exist obvious gaps in the biosynthetic pathways and regulatory mechanisms.

Moreover, the transcriptional regulation of the ester synthesis in kiwifruit, as well as most other fruit, has not been reported. At present, research on the transcriptional regulatory mechanism of fruit aroma production mainly concentrate on terpenes, including citrus *CitAP2.10* for (+)-valencene and *CitERF71* for *E*-geraniol, and kiwifruit *AaNAC2/3/4* for monoterpene biosynthesis [16–18]. In persimmon fruit, the *ERFs* could regulate the *ADH* promoter and might be key components in persimmon fruit astringency removal [19]. In kiwifruit, *AcNAC5* increased significantly in response to propylene and showed correlation with aroma volatile production patterns, suggesting its potential role in the regulation of *AcAAT* and aroma volatile biosynthesis during ripening [3]; However, such findings only hint at the potential of TFs in ester related pathway regulation, and role of these ERFs and other TFs on esters biosynthesis remains unclear. In our previous research, three major ripening traits in kiwifruit, including texture, ethylene production and starch degradation were analyzed and a range of ethylene responsive TFs were identified [20]. Only AdDof3, AdDof4 (DNA binding with one finger) and AdNAC5 were identified as targeting genes related to kiwifruit ripening and softening [20], however, and it was assumed that the remaining TFs may also be involved in other regulating other ripening related traits such as aroma.

Based on the academic background of ester biosynthesis in kiwifruit, the objectives of this research were to identify the key genes for ester production and the underlying transcriptional regulatory mechanisms contributing to the control of their expression. The volatile compounds were quantified by GC-MS, using ethylene treated, 1-MCP treated and control fruit. Three key structural genes were predicted by transcriptomic results, by using Weighted Gene Co-Expression Network Analysis (WGCNA). Furthermore, AdNAC5 and AdDof4 were provisionally identified as transcriptional regulator active on the *AdFAD1* promoter. Thus, these results not only filled in some of the gaps in the ester biosynthetic pathway in kiwifruit, but also identified TFs involved in the regulatory mechanisms of aroma production.

## Results

### Volatile compounds analysis in ripening kiwifruit

**Fig. 1:**
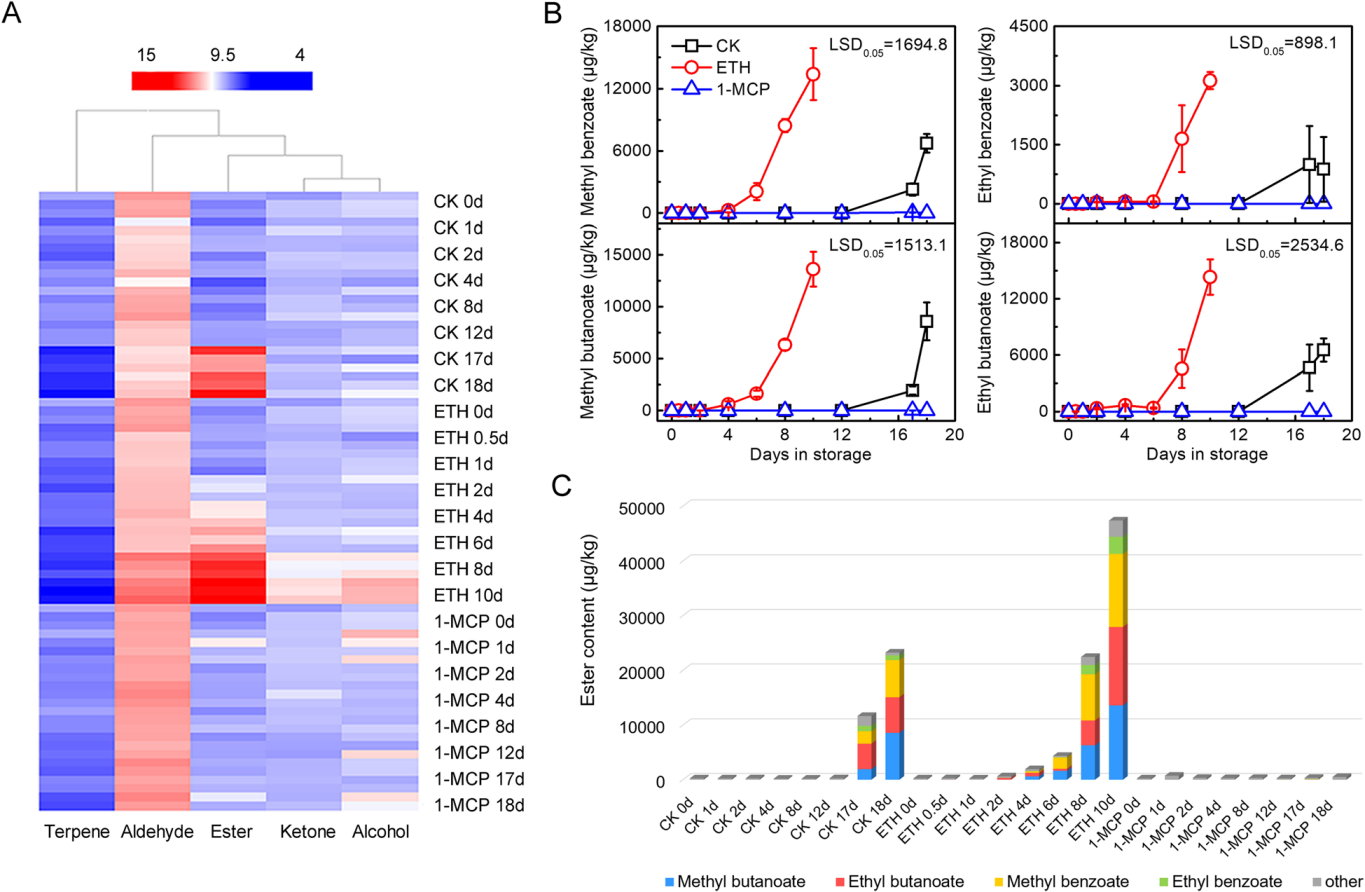
Analysis of volatile compounds in ‘Hayward’ kiwifruit. **a** Total alcohols, terpenes, aldehydes, ketones and esters of kiwifruit in response to ethylene or 1-MCP treatment during storage. Mature fruit were treated with 100 μL L^− 1^ ethylene (ETH), 1 μL L^− 1^ 1-MCP, or air (control, CK) for 24 h at 20 °C. **b** Profiles of methyl butanoate, methyl benzoate, ethyl butanoate, and ethyl benzoate in flesh tissues of ripening kiwifruit. Error bars indicate S.E.s from three replicates. LSD values represent least significant difference at *P =* 0.05. **c** The proportion of methyl butanoate, methyl benzoate, ethyl butanoate, ethyl benzoate and other ester components in total esters

Alcohols, terpenes, aldehydes, ketones and esters were analyzed in the postharvest kiwifruit treated with ethylene, 1-MCP or control, respectively. Among total alcohols, terpenes, aldehydes, ketones and esters, esters were the most numerous compounds present at the ripening stage, with the values increasing from 236.23 μg/kg at 0 d and reaching peaks of 47,373.07 μg/kg in ETH (10 d), 23,232.26 in control (18 d) and 499.82 μg/kg in 1-MCP treated samples (18 d), respectively (Fig. 1a). Concentrations of main volatile compounds can be found in Additional file 1. (*E*)-2-Hexenal stayed high level in most unripen fruit and identified as the major contributors to kiwifruit aroma with green notes [21]. Besides, methyl butanoate, methyl benzoate, ethyl butanoate and ethyl benzoate were the most abundant compounds in ripen fruit (Additional file 2: Figure S1). During storage, these four esters showed similar changes: induced by ethylene and repressed by 1-MCP. The production of esters increased sharply in control (17 d, 18 d) and ETH fruit (8 d, 10 d), concomitantly with endogenous climacteric ethylene production, while they all remained at the basal level throughout storage in 1-MCP treated fruit (Fig. 1b). Other volatiles contributed much less to the aroma profile, compared with these four esters (Fig. 1c). PCA analysis showed that esters compounds located the nearest with ethylene, suggesting the strongly correlations between them and the possibility of esters associated with kiwifruit ripening (Additional file 2: Figure S2).

### WGCNA of RNA-seq data

**Fig. 2:**
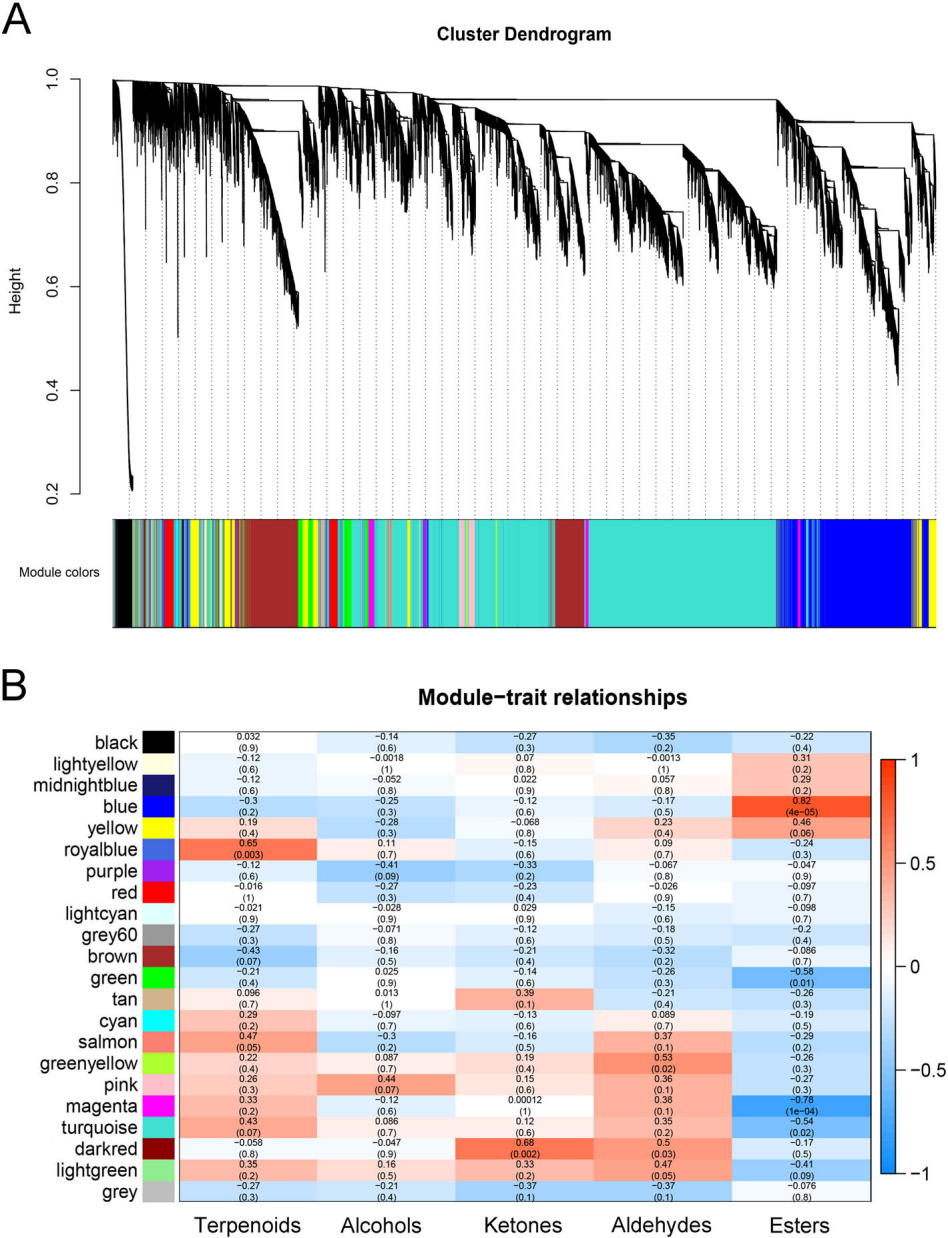
Co-expression network analysis of transcriptomes in relation to aroma contents during ‘Hayward’ kiwifruit fruit ripening. **a** Hierarchical cluster tree showing 22 modules of co-expressed genes. Each leaf represents one gene in the tree. **b** Module-flavor correlations and corresponding *p*-values. Each row corresponds to a cluster. Each column corresponds to a volatile compound. The left panel shows 22 modules and the right panel is a color scale for module trait correlation from − 1 to 1

The WGCNA was performed to investigate the co-expression networks, in which all co-expressed genes were connected to each other with varying correlation strengths. Genes were partitioned into twenty-two co-expression modules (Fig. 2a). The content of esters was positively correlated with gene expression in the ‘blue’ module and negatively correlated in the ‘magenta’ module, with a coefficient of 0.82 (*P* = 4 × 10^− 5^) and − 0.78 (*P* = 1 × 10^− 4^), respectively (Fig. 2b). The coefficients for modules with the other four types of volatile compounds were all lower than 0.7 (Fig. 2b). Based on correlation (*r* > 0.7) between genes in the two modules and ester content, a total of 1896 genes were identified.

**Fig. 3:**
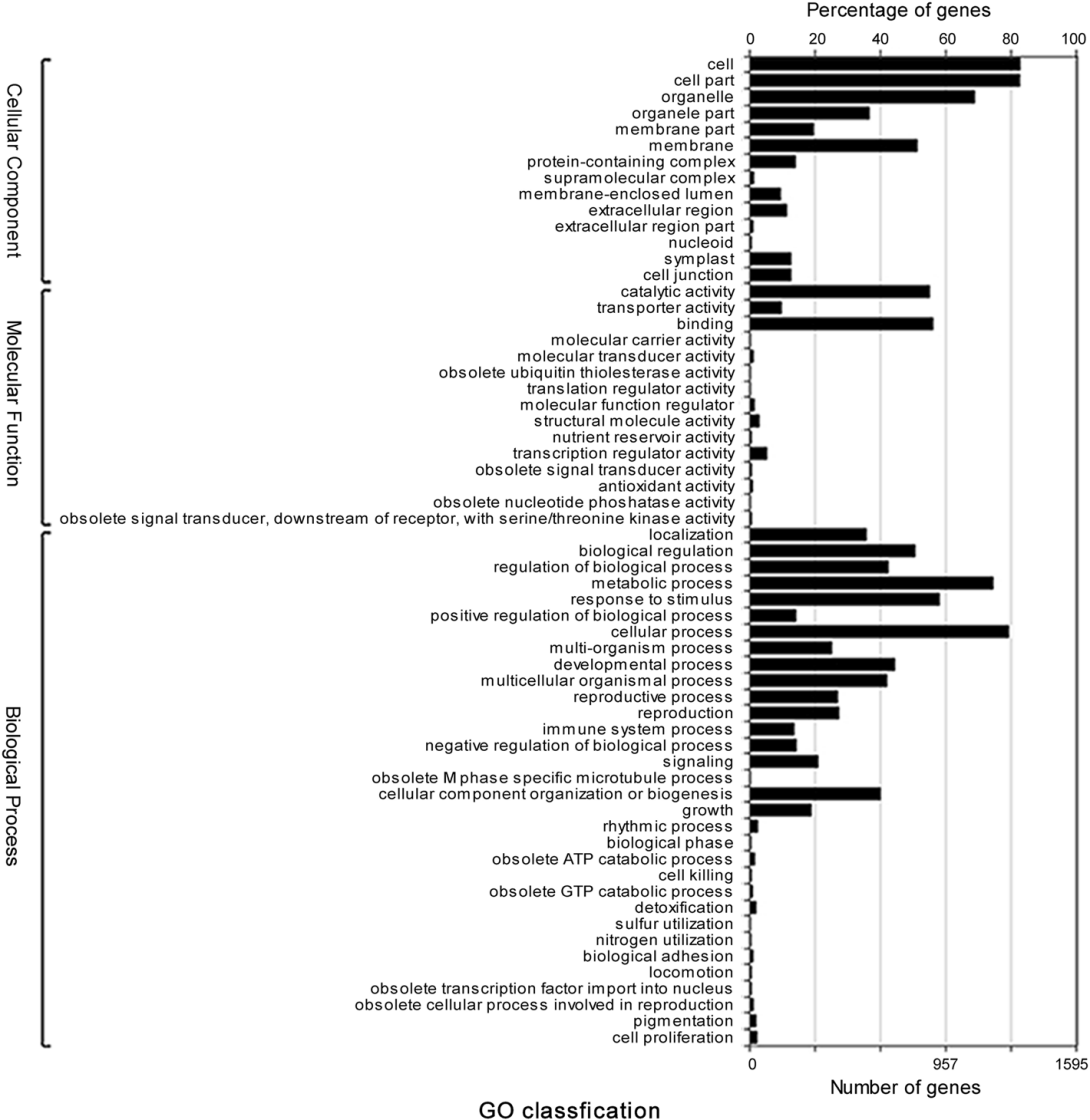
GO annotation of co-expressed genes

Using the 1896 genes, GO annotation was carried out. GO annotation analysis revealed that genes could be summarized in three main functional categories, including cellular component, molecular function and biological process. Five groups, including cell, cell part, cellular process, metabolic process and organelle, were the main classifications for more than 60% of the genes (Fig. 3).

### Identification and expression of ester biosynthesis genes during kiwifruit ripening

**Fig. 4:**
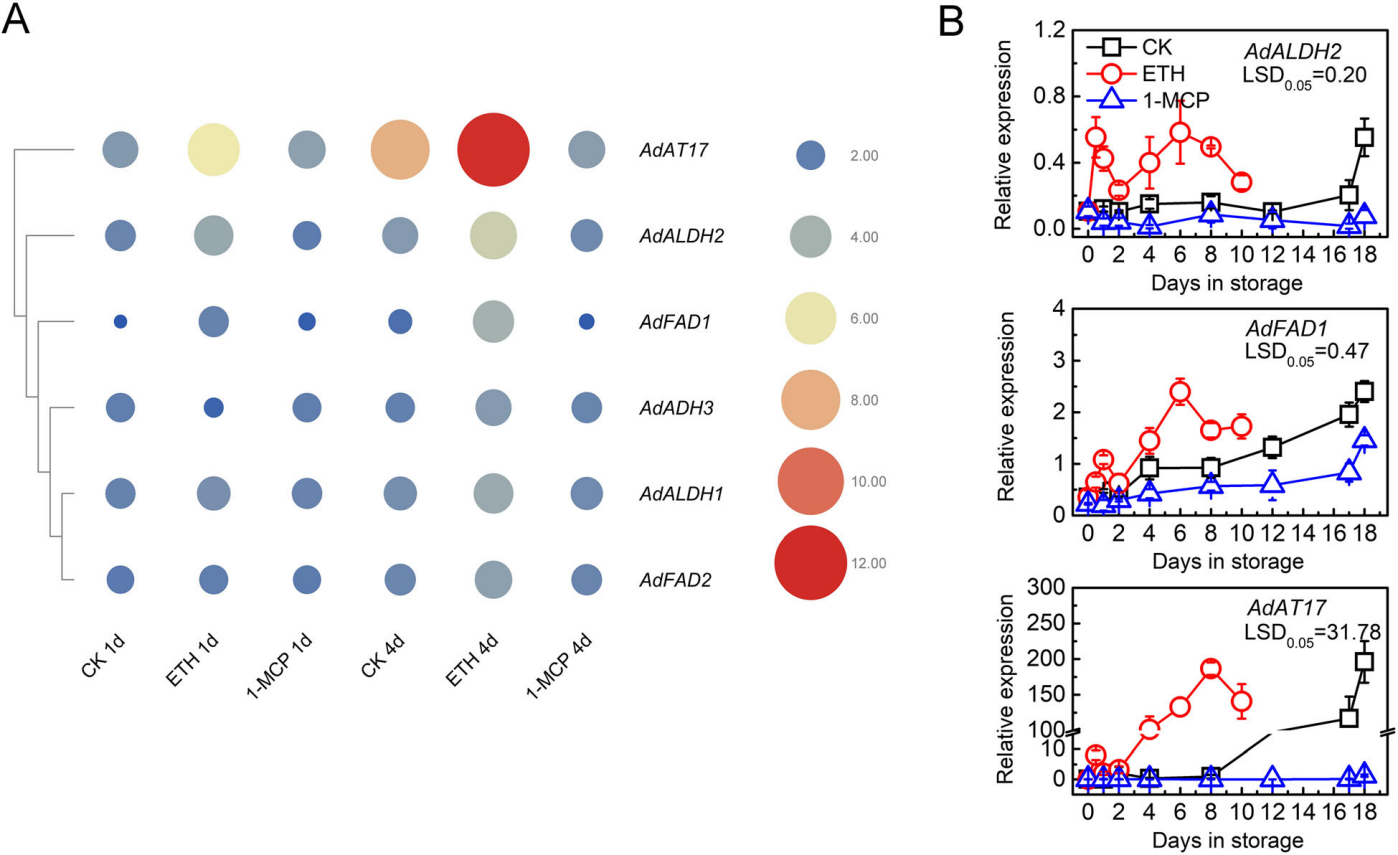
Expression of structural genes in response to ethylene or 1-MCP treatment during ‘Hayward’ kiwifruit storage. Mature fruit were treated with 100 μL L^− 1^ ethylene (ETH), 1 μL L^− 1^ 1-MCP, or air (control, CK) for 24 h at 20 °C. **a** Structural genes with putative function in kiwifruit ester biosynthesis from RNA-seq. Transcript abundance is indicated by color and size, the bigger and redder the circle, the higher is the expression. Mean data was obtained from three replicates. **b** Gene expression was analyzed by real-time PCR. Error bars represent S.E.s from three replications. LSD values represent least significant difference at *P =* 0.05

Based on WGCNA narrowed the scope of differentially expressed genes (DEGs) from RNA-seq and focused on biosynthetic pathways for esters, two aldehyde dehydrogenases (*AdALDH1*, *AdALDH2*), two fatty acid desaturases (*AdFAD1*, *AdFAD2*), one alcohol dehydrogenase (*AdADH3*) and one alcohol acyltransferase (*AdAT17*) [22] were identified. Using TBTools software, gene transcripts abundance is indicated with a heatmap (Fig. 4a). Among the genes, *AdAT17*, *AdALDH2* and *AdFAD1* exhibited similar pattern, which were obviously induced by exogenous ethylene and suppressed by 1-MCP treatment, while *AdADH3*, *AdALDH1* and *AdFAD2* were less responsive to two treatments (Fig. 4a). Increases in abundance of transcripts of *AdAT17*, *AdALDH2* and *AdFAD1* were confirmed by real-time PCR in postharvest ‘Hayward’ kiwifruit. The results indicated that *AdAT17*, *AdALDH2* and *AdFAD1* were all responsive to exogenous ethylene at 1 d and were also accumulated in parallel with endogenous ethylene in both ETH and control fruit (Fig. 4b). 1-MCP treatment kept expression of *AdAT17* and *AdALDH2* at basal level, while it only delayed the increase in expression of *AdFAD1* (Fig. 4b). The most significant difference was observed for *AdAT17*, its relative expression reaching the maximum (186.53) at 8 d in ETH, compared to 0.93 for CK and 0.05 for 1-MCP at 8 d (Fig. 4).

### Regulatory effects of transcription factors on two main ester biosynthesis genes (*AdFAD1* and *AdAT17*)

**Fig. 5:**
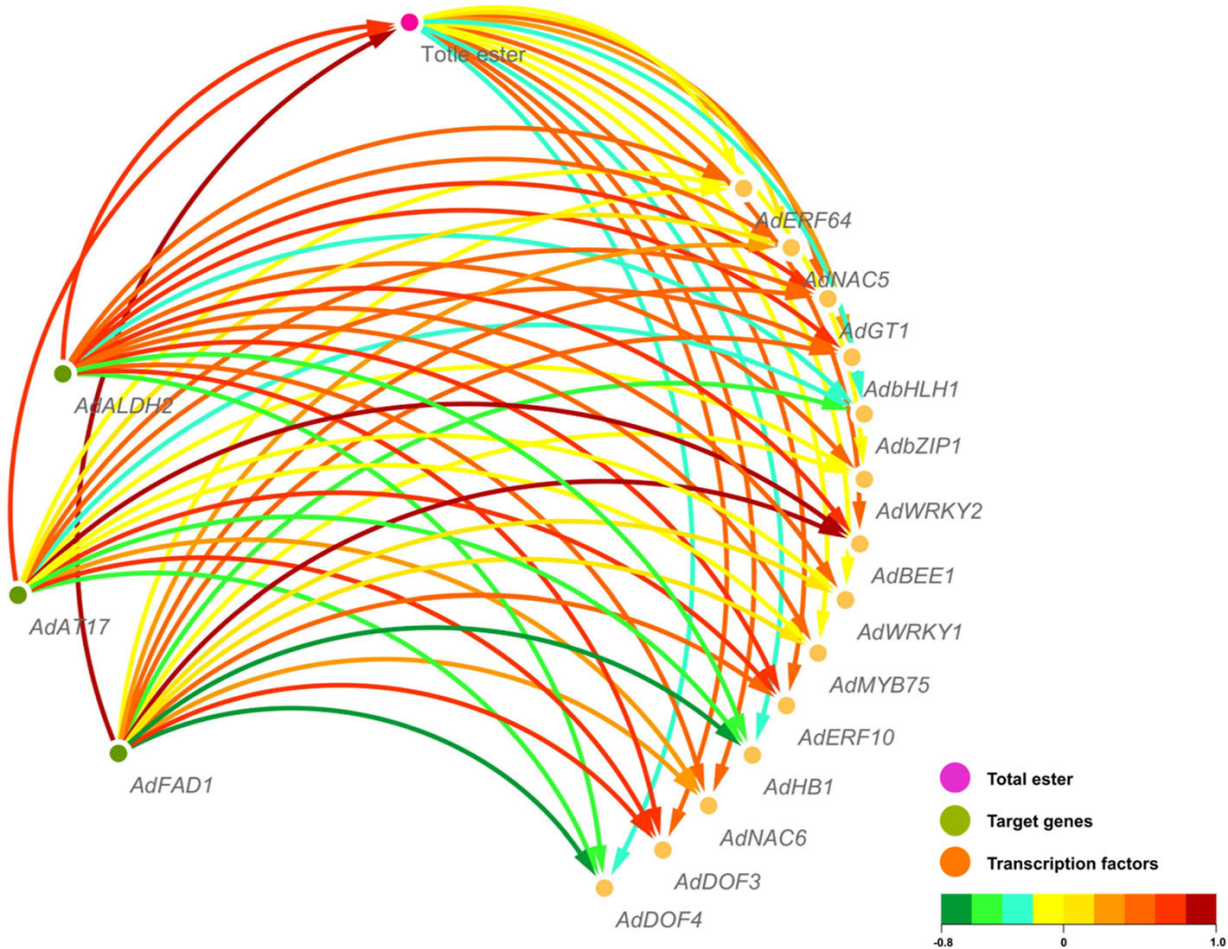
Correlation network analysis for structural genes, transcription factors and esters content. The colors of arrows represent the correlations, as indicated by colored bar

Correlation analysis with ester content, and three candidate genes (*AdFAD1*, *AdAT17* and *AdALDH2*) potentially being involved in ester biosynthesis with 14 previously characterized ripening related TFs [20], were conducted and visualized by Cytoscape (v3.7.1, USA). As shown in Fig. 5, all three ester biosynthesis genes exhibit positive correlations with ester content, with *AdFAD1* showing the highest positive correlation. For the 14 TFs, the transcript changes in *AdBEE1*, *AdERF10*, *AdNAC6*, *AdGT1*, *AdDof3* and *AdNAC5* positively correlated with ester content, while *AdDof4*, *AdHB1* and *AdbZIP1* showed negative correlations (Fig. 5). Between ester biosynthesis genes and TFs, the highest positive correlation was observed between *AdBEE1* and *AdAT17*, followed by *AdBEE1* and *AdFAD1*, while the highest negative correlation was found between *AdDof4* and *AdFAD1* (Fig. 5).

**Fig. 6:**
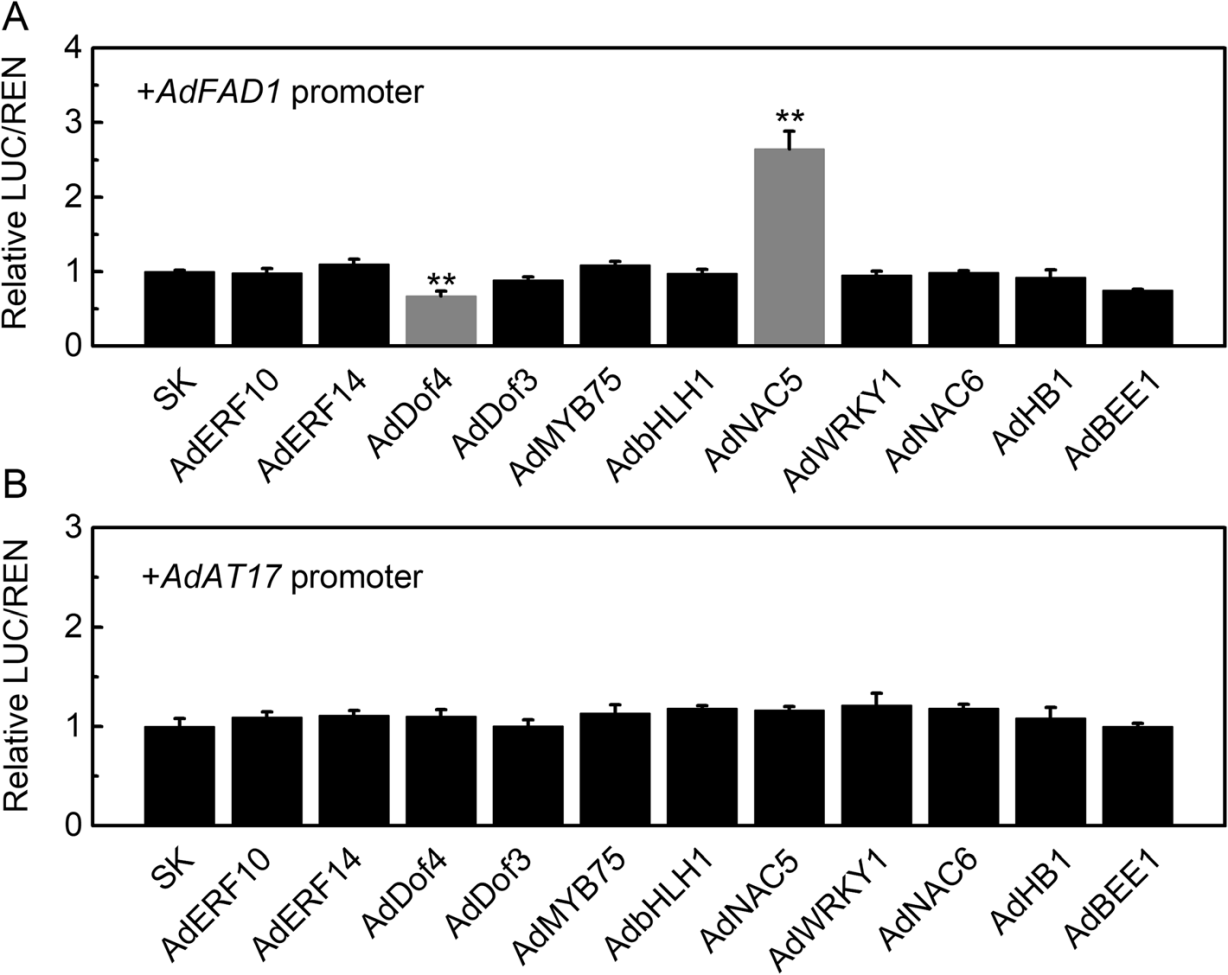
Regulatory effects of transcription factors on promoters of *AdFAD1* (**a**) and *AdAT17* (**b**), by dual-luciferase assays. The ratio of LUC/REN of the empty vector plus promoter was set as 1. SK refers to the empty pGreen II 0029 62-SK vector. Error bars indicate S.E.s from three replicates (*P < 0.05, **P < 0.01 and ***P < 0.001)

To extend beyond the digital analysis of the relationship between TFs, structural genes, and ester content, the in vivo regulatory roles of TFs on the promoters of two of the main candidate targets (*AdAT17* and *AdFAD1*) were tested, using dual-luciferase assay. Unfortunately, the failure of amplification of the *AdALDH2* promoter precluded its inclusion in this analysis. The results indicated that AdNAC5 could significantly trans-activate the *AdFAD1* promoter, with over 2-fold induction (Fig. 6a), whereas for the *AdFAD1* promoter, AdDof4 acted as a repressor, reducing the transcriptional activity to 0.67, compared to the basal activity set as 1 (Fig. 6a). None of the examined TFs showed significant regulatory effects (neither activation nor repression) on the *AdAT17* promoter, (Fig. 6b).

## Discussion

### Regulatory roles of ethylene and 1-MCP on esters accumulation

Understanding aroma formation is critical for kiwifruit quality and marketability, especially the production of esters, which are the main components for aroma in ripening kiwifruit (Fig. 1a). During kiwifruit ripening, the flavor changes from ‘grassy’ and ‘green’ to ‘fruity’, and this is manifested by increases in esters and decreases in aldehydes [21]. It was suggested that the accumulation of esters during ripening were likely driven by ethylene [8, 12]. The production of esters, especially ethyl butanoate and methyl butanoate, appeared to be strongly dependent on ethylene signal [3]. Additionally, in ethylene-suppressed transgenic lines kiwifruit, esters were dramatically reduced, while the major volatiles produced in ethylene-unsuppressed fruit were ethyl butanoate, methyl butanoate, and ethyl acetate [4]. The present study also found that ethylene treatment accelerated esters accumulation compared with control fruit, and 1-MCP treatment showed the opposite effects on ester production. Four ester compounds (methyl butanoate, methyl benzoate, ethyl butanoate and ethyl benzoate) were found as the main contributors to the total ester for ripening ‘Hayward’ kiwifruit (Fig. 1c), which were similar to previous findings [8, 9]. During ‘Hayward’ storage at 0 °C, the total esters, which consisted mainly of methyl and ethyl butanoate, increased after 9 weeks storage but dropped after 21 weeks storage, and methyl and ethyl benzoate showed similar trends [8]. Butanoates were the main fruity esters in both ‘Hayward’ and ‘Hort16A’ fruit, which significantly increased during ripening and in over-ripe fruit. Butanoates contributed to less than 40% of the esters in ‘Hayward’ fruit but greater than 60% in ‘Hort16A’ [9]. Zhang et al. reported ethyl and methyl butanoate levels increased late in the ripening process of ‘Bruno’ [12]. In ‘Hort16A’ kiwifruit, ethylene treatment (100 ppm, 24 h) can restore the significant reduction of aroma-related esters by cold storage (1.5 °C) for 2 or 4 months [23]. Moreover, the effects of ethylene and/or 1-MCP on esters has also been widely reported in other fruit. In pear fruit, concentrations of both total volatiles and esters were lower in 1-MCP treated fruit compared with control [24]. In apple fruit, regulation of ethylene has a major effect on volatiles production, AAT enzyme is an important point in the modulation of ester production under ethylene regulation [25]. Furthermore, ethylene is also known to be important for the expression of some volatile-related genes in melon and tomato [26, 27].

The impact of ethylene on ester production in climacteric fruit is well accepted. Thus, at the physiology level, we recorded the changes in aroma compounds during the whole storage and maturation period. The present findings confirmed a burgeoning increase in esters content of ‘Hayward’ coinciding with fruit softening, which was accelerated by ethylene treatment and delayed by the ethylene action inhibitor 1-MCP. These results directly conform the important impact of ethylene for ester biosynthesis regulation.

### Candidate target genes for ester biosynthesis

Beyond the physiology studies, the molecular aspects underlying ester biosynthesis were also widely studied. Yahyaoui et al. showed that AAT was involved in the generation of aroma volatile esters during melon ripening [26]. Transgenic apple plants with reduced *MpAAT1* expression showed reduced levels of most key esters [22]. *MdAAT2* was also positively correlated with AAT enzyme activity and ester production in ‘Golden Delicious’ [28]. Qian et al. demonstrated the *AAT* gene mainly determined the contents of berry volatile esters [29]. Balbontin et al. indicated that papaya fruit *VpAAT1* encoded a functional enzyme with high AAT activity, contributing to the formation of benzyl acetate, and its expression was dependent on ethylene [30]. Cumplido-Laso et al. observed a significant reduction in ester production in the strawberry fruit receptacle when *FaAAT2* expression was transiently downregulated [31]. Besides AAT, there are multiple other enzymes and their coding genes involved in ester production, such as fatty acid desaturase (FAD), alcohol dehydrogenase (ADH), lipoxygenase (LOX). In peach fruit, HA (hot air) and UV-C treatments effectively promoted the emission of esters, which were consistent with up-regulations on *PpAAT*, *PpFAD* and *PpACX1/2* expression [32]. In pear fruit, *PuLOXs* and *PuAAT* were inhibited dramatically by 1-MCP, and the suppression of *PuLOXs* and *PuAAT* genes may be ascribed to the reason for lower content of total volatiles and esters [24]. Moreover, overexpression of *FAD* (*FAD3* and *FAD7*) in tomato fruit promoted linolenic acid content and enhanced an accumulation of volatiles [33]. In tomato, LOXC has been shown to be involved directly in the production of aroma volatiles [27]. In kiwifruit, the enzymes and their coding genes for ester were mainly focused on lipoxygenase (LOX) and alcohol acyltransferase (AAT). The activity of LOX enzyme tended to increase with fruit ripening and were up-regulated by ethylene treatment [11, 12], although in tomato ethylene stimulates accumulation of transcripts from some *LOX* genes, including *TomLoxc* involved in flavor volatile production, and suppresses other *LOX* genes [27]. The activity of AAT enzyme is exclusive to ethylene-producing kiwifruit and the production of esters depends on ethylene-regulated AAT enzyme levels [13]. As for the coding genes, six *LOX* genes (*AdLox1–6*) and 12 *AATs* (*AdAT1*/*2*/*6*/*17*/*22*, *AeAT9*, *AcAT15*/*16*/*20*/*23*/*24*, *AaAT18*) were identified, and *AdLox1*, *AdLox5* and 12 *AATs* were thought to be potential candidates for flavor-related esters synthesis [11, 12, 14]. Günther et al. confirmed that accumulation of *AAT1*, *AAT2*, *AAT15*, *AAT16*, *AAT17* and *AAT18* transcripts is dependent on ethylene-induced ripening [13]. *AcAAT* expression exhibited a dramatic increase in propylene treated kiwifruit [3]. The present transcriptome-based analysis and WGCNA analysis indicated three key genes (*AdALDH2*, *AdFAD1* and *AdAT17*) are critically important for kiwifruit ester production, and highlights the importance of these genes for understanding ester metabolism in kiwifruit. Phylogenetic analysis indicated that *AdAT17* clustered with flavor-related *AATs* from other plant species, including *FaAAT2*, *VpAAT1*, *CmAAT1–3* and apple *AAT1*–*4* (Additional file 2: Figure S3), while *AdFAD1* clustered closest with *AtFAD2* (Additional file 2: Figure S4), which acts on fatty acids in conjunction with the activity of the ER 18:1 desaturase [34].

### The proposed transcriptional mechanism for fruit ester biosynthesis

Beyond the biosynthesis pathway, there is very little information about the TFs involved in transcriptional control of ester metabolism and recent investigations have mainly been conducted on terpene biosynthesis. For instance, kiwifruit *AaNAC2/3/4* are involved in the transcriptional regulation of *TPS* genes (*AaTPS1*) and monoterpene production [18]; citrus CitAP2.10 and CitERF71 regulate corresponding *TPS* genes (*CsTPS1* and *CitTPS16*) and contributed to (+)-valencene and *E*-geraniol metabolism, respectively [16, 17]. Moreover, in strawberry, an ERF-MYB transcription complex was found to regulate furaneol biosynthesis and the *FaQR* promoter [35]. However, few TFs have been reported that have regulatory effects on biosynthesis of esters. The present finding indicated that one of the key biosynthetic genes (*AdFAD1*) could be activated by AdNAC5 and suppressed by AdDof4 (Fig. 6), which provided an elementary example of a transcriptional regulatory module for esters metabolism. NAC transcription factors (*AaNAC2/3/4*) are involved in kiwifruit terpene biosynthesis [18], however, the phylogenetic tree aligned the *AdNAC5* sequence with the reported *NACs* in *A. chinensis* and *A. arguta* and *AdNAC5* does not have high homology to the kiwifruit NACs involved in terpene biosynthesis and the more closely related genes (AT5G61430.1, AT5G07680.1 and AT3G18400.1) in *Arabidopsis thaliana* are all functionally uncharacterized (Additional file 2: Figure S5).

In plants, Dof transcription factors have been widely reported to be involved in many biological processes, such as sweet potato *SRF1*, maize *Zmdof3* and kiwifruit *AdDof3* that participate in regulating carbohydrate and starch metabolism [20, 36, 37]. Banana MaDof23 can interact with MaERF9 in regulating fruit ripening [38], however, the roles of Dof transcription factors in aroma production have rarely been investigated. Thus, the regulatory roles of NAC and Dof on kiwifruit ester-related genes not only provide new information about fruit aroma (especially for ester biosynthesis) regulation, but also have suggested the novel transcription factors that may contribute volatile metabolism.

## Conclusions

In summary, using GC-MS the present study indicated the role of the four main esters (methyl butanoate, methyl benzoate, ethyl butanoate and ethyl benzoate) in ripening ‘Hayward’ kiwifruit. Further investigation based on RNA-seq and WGCNA, suggested the three key structural genes (*AdFAD1*, *AdAT17* and *AdALDH2*) for ester production. Furthermore, testing the role of TFs in transcriptional regulation characterized AdNAC5 as a transcriptional activator and AdDof4 as a transcriptional repressor of the *AdFAD1* promoter. Overall, these findings provide new insights into the production of volatiles in ripening kiwifruit and the roles of specific structural genes and TFs in ester metabolism.

## Methods

### Plant material and treatments

The mature kiwifruit (*Actinidia deliciosa* [A. Chev.] C.F. Liang et A.R. Ferguson var. *deliciosa* cv. Hayward) were harvested in the 2015 season from a commercial orchard (Shanxi, China), with a mean total soluble solid (TSS) of 6.19%. Kiwifruit ‘Hayward’ is a breeding plant material originally from New Zealand and available at Wuhan Botanical Garden, Chinese Academy of Sciences. We declare that the kiwifruit materials in this study comply with institutional, national, and international guidelines for the collection and cultivation of any plant materials. Fruit of uniform size without visible defects were divided into three batches. One batch was treated with C_2_H_4_ (ETH, 100 μl l^− 1^, 24 h, 20 °C), one was treated with 1-MCP (1 μl l^− 1^, 24 h, 20 °C) and the third batch was sealed in air as control (air, 24 h, 20 °C) and they were all placed at 20 °C. Each treatment contained three biological replicates of approximately 200 fruit. At each sampling point, three replicates of four fruit were collected from each batch. Samples were taken at intervals and the materials (small pieces of outer pericarp free of skin or seeds) were frozen with liquid nitrogen and stored at − 80 °C. The treatment and sample collection, as well as the ripening related indexes (firmness, cell wall materials, starch, TSS, ethylene) were conducted as described by Zhang et al [20]*.*

### Volatile analysis by GC-MS

Measurement of postharvest kiwifruit volatiles and analysis were carried out according to Zhang et al. and Wu et al [12, 39]. Frozen flesh tissue was ground in liquid nitrogen and a total of 2 g flesh transferred to a 10 mL vial containing 3 mL saturated sodium chloride solution. Each sample point had three replicates. Before the vials were sealed, 10 μL of 2-octanol (0.8 mg/mL) was added as the internal standard. After vigorous vortexing, samples were equilibrated at 45 °C for 30 min and volatiles collected with a solid-phase microextraction (SPME) fiber coated with a 65 μm of polydimethylsiloxane and divinylbenzene (PDMS-DVB) (Supelco Inc., Bellefonte, PA, USA). The volatile compounds were identified using an Agilent 7890 N gas chromatograph coupled with an Agilent 5975C mass spectrophotometer (Agilent, Palo Alto, CA, USA). The Agilent 5975C mass spectrophotometer was equipped with a DB-WAX column (30 m, 0.32 mm, 0.25 μm, J&W Scientific, Folsom, CA, USA). The oven temperature program started at 40 °C and increased to 100 °C at a rate of 3 °C min^− 1^, followed by a ramp to 245 °C at a rate of 5 °C min^− 1^. Helium was used as carrier gas at the flow rate of 1.0 ml min^− 1^. The source temperature for the MS was 230 °C, and the column effluent was ionized by electron energy of 70 eV with the transfer interface zone at 250 °C. The mass scanning was done over the range of 35–350 m/z. Identification of volatile compounds was further validated by comparing their electron ionization mass spectra with the NIST/EPA/NIH Mass Spectral Library (NIST-08) and the retention time of authentic standards.

### RNA extraction and cDNA synthesis

Total RNA was isolated from frozen kiwifruit flesh following the protocol described by Yin et al [40]*.* From each sample, 1 μg of total RNA in a 20 μL solution was reverse transcribed using the PrimeScript 1st Strand cDNA synthesis kit (TaKaRa, Dalian, China) following the manufacturer’s protocol. At each sampling point, RNA extraction and cDNA synthesis were performed with three biological replicates.

### RNA-seq, WGCNA and gene network visualization

The details of RNA-seq were described by Zhang et al [20]. The SRA accession number are SRR6885590-SRR6885601, SRR7630964-SRR7630969.

Co-expression networks were created using WGCNA (v1.29) package in R [41]. The data used for WGCNA co-expression network analysis were the five classes of aroma components (terpenes, alcohols, aldehydes, ketones and esters) produced during storage and all RNA-seq genes after discarding none detectable genes (FPKM = 0). A total of 26,111 genes were used as input to the signed WGCNA network construction. The automatic network construction function block wise was used to build modules. In standard WGCNA networks, soft power was set to 6, min Module Size was 30, and the merge Cut Height value was 0.25. The initial clusters were merged on eigengenes. Eigengene value was calculated for each module, which was used to search the association with flavor compounds. WGCNA is accomplished using a soft threshold to preserve the continuous nature of the data set and eliminate the need to set an arbitrary correlation score cutoff. Candidate hub genes in “blue” and “magenta” were picked by threshold at a value of 0.7. The network of ester content, transcription factors and candidate target genes were visualized by Cytoscape (v3.7.1, USA).

### Real-time PCR analysis

Real-time PCR was carried out with a LightCycler® 480 SYBR Green I Master (Roche) kit using a LightCycler® 480 instrument (Roche). The information about structural genes is listed in Additional file 3: Table S1. The primers for real-time PCR analysis were designed by primer3 (http://frodo.wi.mit.edu/primer3) and are listed in Additional file 3: Table S2. The specificity of primers was double checked by melting curve and PCR product resequencing described by Yin et al [40]*.* Abundance of cDNA templates was monitored with kiwifruit actin, which is stably expressed across kiwifruit ripening stages [11]. Three biological replicates were analyzed for real-time PCR. The PCR program was initiated with a preliminary step of 5 min at 95 °C, followed by 45 cycles at 95 °C for 10 s, 60 °C for 10 s, and 72 °C for 15 s. Data were analyzed with the 2^-∆Ct^ method to calculate the relative expression levels of genes, by transcript abundance.

### Gene isolation and promoter cloning

Based on the RNA-Seq results, differentially expressed genes (DEGs) associated with ester biosynthesis were isolated and verified by PCR with gene-specific primers (Additional file 3: Table S2) using cDNA from cv. ‘Hayward’. The transcription factors were derived from our previous report [20].

Using the RNA-Seq results and kiwifruit genome database for reference [42], the promoter sequences were amplified (Additional file 3: Table S3). Genomic DNA (gDNA) from ‘Hayward’ served as template for amplifying *AdAT17* and *AdFAD1* promoters. The primers are listed in Additional file 3: Table S4.

### Dual-luciferase assays

Full length sequences of eleven transcription factors were integrated into pGreen II 0029 62-SK vector (SK) [20], while promoters of *AdAT17* and *AdFAD1* were inserted into the pGreen II 0800-LUC vector (LUC).

The recombinant SK and LUC vectors were transfected into *Agrobacterium tumefaciens* GV3101 and stored at − 80 °C with glycerol. Before injection, the glycerol stocks were activated on new LB plates to which was added 50 μg ml^− 1^ kanamycin and 25 μg ml^− 1^ gentamycin. Then the *Agrobacterium* with recombinant vectors were suspended in infiltration buffer (10 mM MgCl_2_, 10 mM MES, 150 mM acetosyringone, pH 5.6) to the optimal density (OD_600_~0.75). After that, 100 μl of *Agrobacterium* containing promoters were mixed with 1 ml of *Agrobacterium* cultures containing transcription factors, then were injected into tobacco (*Nicotiana tabacum*) leaves using needleless syringes. The empty SK vector was injected as control. Three days after infiltration, the dual luciferase assay reagents (Promega) were used to measure LUC and REN fluorescence intensities following the manufacturer’s instructions. The dual-luciferase assays were performed with three biological replicates.

### Statistical analysis

Results were analyzed by DPS7.05 (Zhejiang University, Hangzhou, China) to compare significant differences, using least significant difference (LSD) at the 5% level. The heatmap was drawn with TBtools software. Figures were drawn with Origin 8.0 (Microcal Software Inc., Northampton, MA, USA). The statistical significance was calculated with a Student’s *t*-test (*P < 0.05, **P < 0.01 and ***P < 0.001).

## Supplementary information

**Additional file 1.** Concentrations of volatile compounds detected in ‘Hayward’ kiwifruit during storage (ng/g FW).

**Additional file 2:Figure S1.** An heatmap of volatile compounds detected in ‘Hayward’ kiwifruit in response to ethylene or 1-MCP treatment during storage. **Figure S2.** PCA analysis of the volitial compounds and ethylene production in kiwifruit treated with control, ethylene (100 μl l^− 1^, 24 h) and 1-MCP (1 μl l^− 1^, 24 h). **Figure S3.** Phylogenetic tree analysis of kiwifruit *AdAT17* and *AAT* sequences in other species. **Figure S4.** Phylogenetic tree of kiwifruit *AdFAD1* with *Arabidopsis thaliana AtFAD2–8*, tomato *SlFAD3*/*StFAD7* and *PpFAD*. **Figure S5.** Phylogenetic tree of AdNAC5 with kiwifruit (*A. chinensis* and *A. arguta*) reported *NACs* and *Arabidopsis thaliana NACs*.

**Additional file 3:Table S1.** Aroma biosynthesis structural genes. **Table S2.** Primers for real-time PCR. **Table S3.** Sequences (5′ to 3′) for promoter isolation. **Table S4.** Primers for vector construction for dual-luciferase assays.

## Data Availability

The raw data have been submitted [20]. The SRA accession number are SRR6885590-SRR6885601, SRR7630964-SRR7630969.

## Abbreviations

1-MCP: 1-methylcyclopropene
AAT: Alcohol acyltransferase
ADH: Alcohol dehydrogenase
ALDH: Aldehyde dehydrogenase
AT: Acyltransferases
d: Day
Dof: DNA binding with one finger
ETH: Ethylene
FAD: Fatty acid desaturase
GC-MS: Gas chromatography-mass spectrometry
HPL: Hydroperoxide lyase
LOX: Lipoxygenas
TFs: Transcription factors
WGCNA: Weighted Gene Co-Expression Network Analysis
