# Supplementary material for: Transcriptome co-expression network analysis identifies key genes and regulators of ripening kiwifruit ester biosynthesis
Source: BMC Plant Biol. 2020 Mar 6;20:103. doi: 10.1186/s12870-020-2314-9 (PMC7059668; doi:10.1186/s12870-020-2314-9)
Supplement: Supplementary file 2 — Additional file 2:Figure S1. An heatmap of volatile compounds detected in ‘Hayward’ kiwifruit in response to ethylene or 1-MCP treatment during storage. Figure S2. PCA analysis of the volitial compounds and ethylene production in kiwifruit treated with control, ethylene (100 μl l− 1, 24 h) and 1-MCP (1 μl l− 1, 24 h). Figure S3. Phylogenetic tree analysis of kiwifruit AdAT17 and AAT sequences in other species. Figure S4. Phylogenetic tree of kiwifruit AdFAD1 with Arabidopsis thaliana AtFAD2–8, tomato SlFAD3/StFAD7 and PpFAD. Figure S5. Phylogenetic tree of AdNAC5 with kiwifruit (A. chinensis and A. arguta) reported NACs and Arabidopsis thaliana NACs. [file 12870_2020_2314_MOESM2_ESM.docx]

**
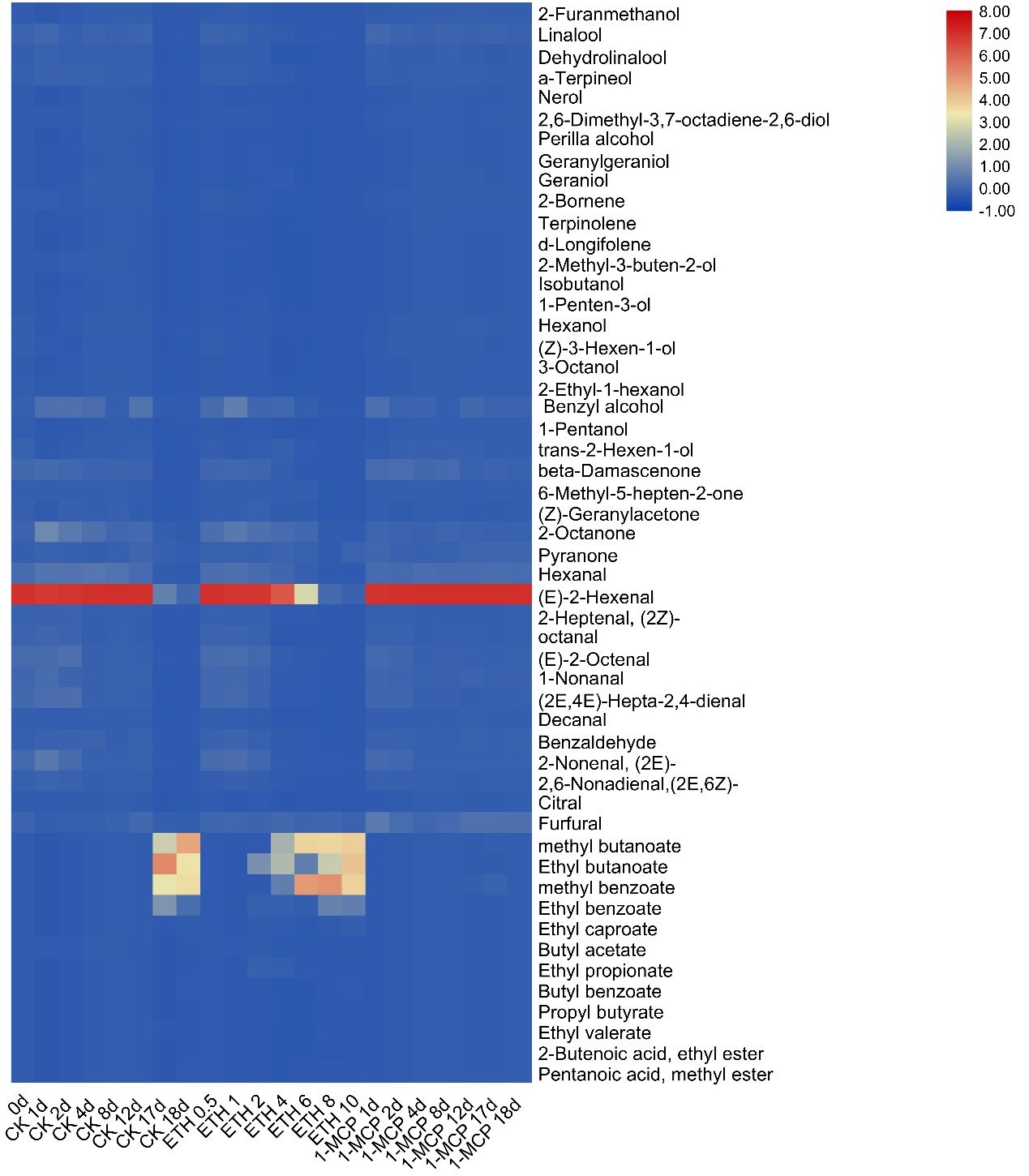
**

**Additional file 2 Fig. S1** An heatmap of volatile compounds detected in ‘Hayward’ kiwifruit in response to ethylene (100 µl l^-1^, 24 h) or 1-MCP (1 µl l^-1^, 24 h) treatment during storage.

**
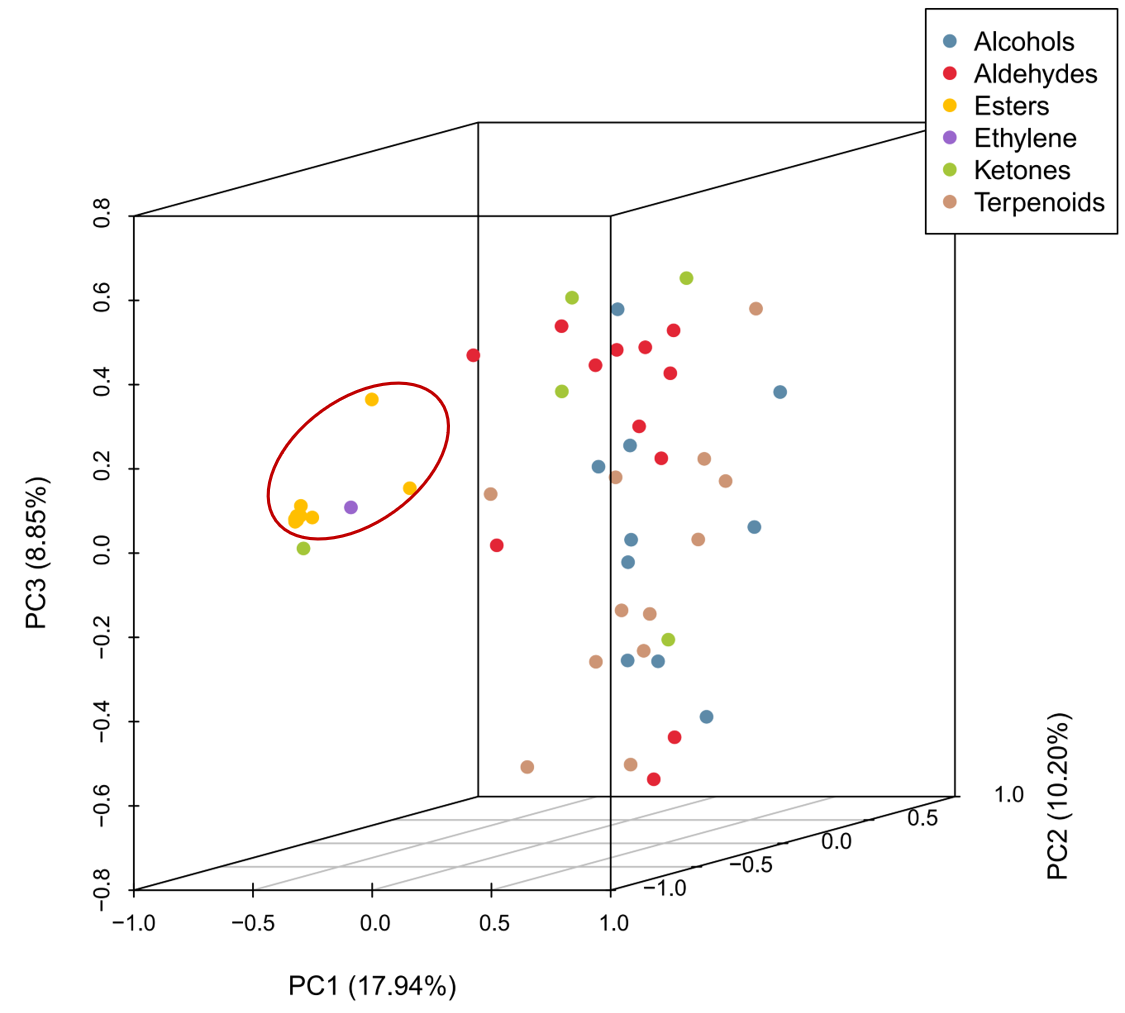
**

**Additional file 2 Fig. S2** PCA analysis of the volitial compounds and ethylene production in kiwifruit treated with control, ethylene (100 µl l^-1^, 24 h) and 1-MCP (1 µl l^-1^, 24 h). The PCA analysis used mean data.

**
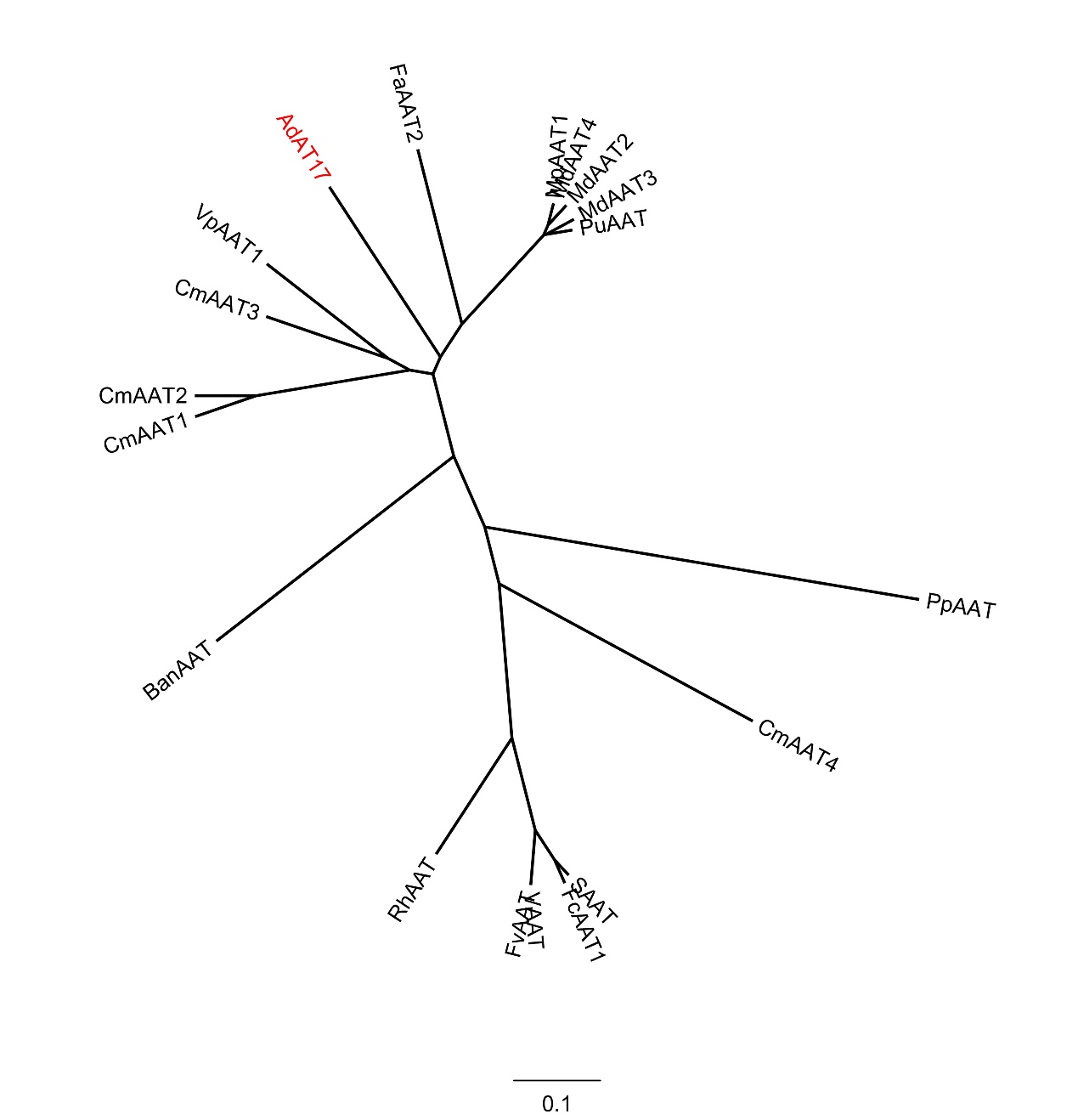
**

**Additional file 2 Fig. S3.** Phylogenetic tree analysis of kiwifruit *AdAT17* and *AAT* sequences in other species. *MpAAT1* *(Malus pumila*, AY707098), *MdAAT2* (*Malus domestica*, AY517491), *MdAAT3* (*Malus domestica*, AY512893), *MdAAT4* (*Malus domestica*, AX025508), *PuAAT* (*Pyrus communis*, AY534530), *BanAAT* (*Musa acuminata*, AX025506), *CmAAT1-4* (*Cucumis melo*, CAA94432, AAL77060, AAW51125, AAW51126), *VpAAT1* (*Vasconcellea pubescens*, FJ548611), *SAAT* (*Fragaria* × *ananassa*, AAG13130), *VAAT* (*Fragaria vesca*, AX025504), *FcAAT1* (*Fragaria Chiloensis*, FJ548610) *FaAAT2* (*Fragaria* × *ananassa*, JN089766), *FvAAT* (*Fragaria* *vesca*, AAN07090), *RhAAT* (*Rosa × hybrida*, AAW31948), *PpAAT* (*Prunus persica*, XM_020562516.1). The phylogenetic trees were constructed using FigTree (v1.4.2).

**
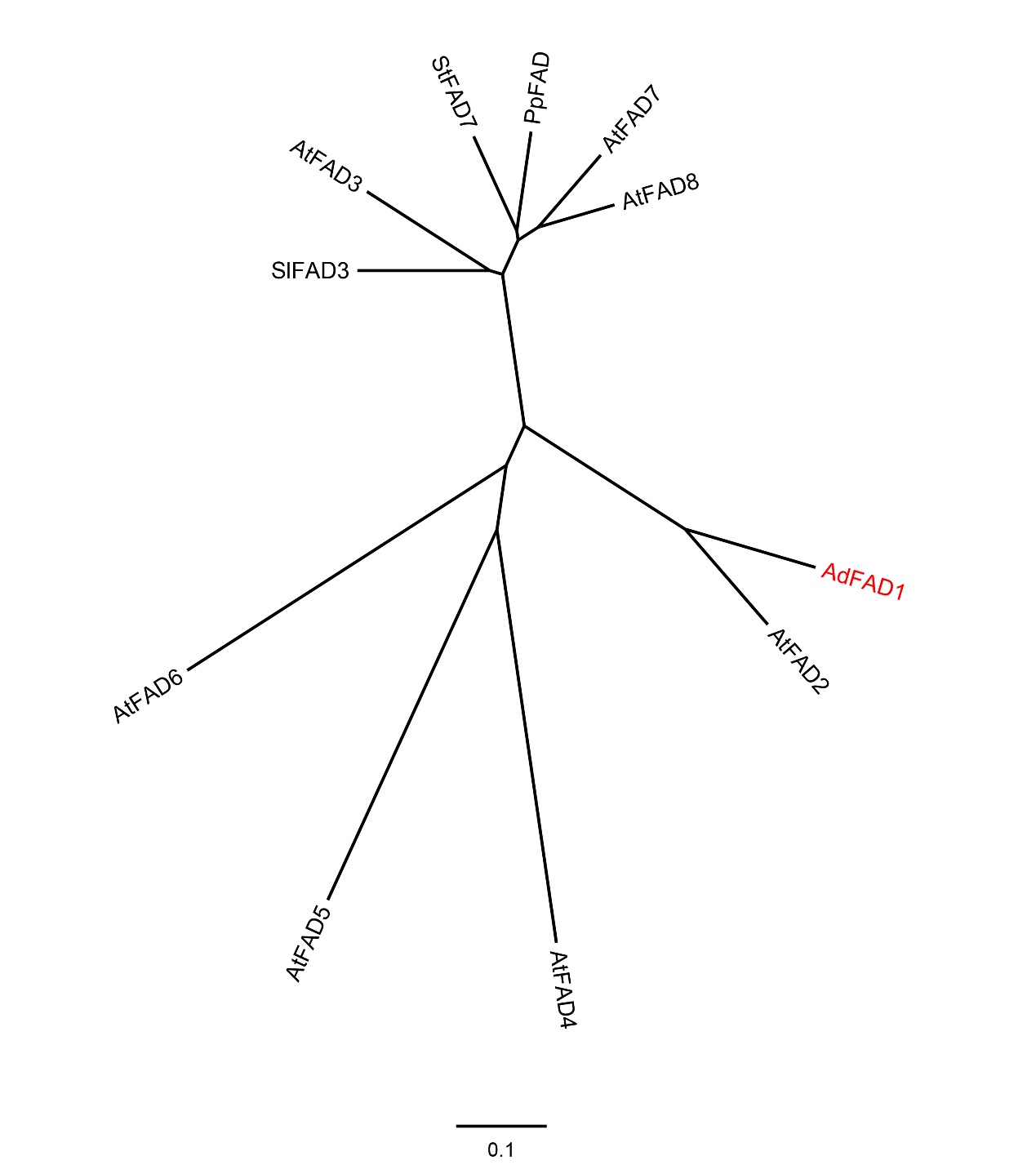
**

**Additional file 2 Fig. S4.** Phylogenetic tree analysis of kiwifruit *AdFAD1* with *Arabidopsis thaliana* *AtFAD2-8*, tomato *SlFAD3* (*Solanum lycopersicum*, EU251190.1) and *StFAD7* (*Solanum tuberosum*, AJ007739.1). *PpFAD* (*Prunus persica*, XM_007217933.2). The deduced amino acid sequences of *Arabidopsis* *thaliana* *FADs* were obtained from TAIR. The phylogenetic trees were constructed using FigTree (v1.4.2).

**
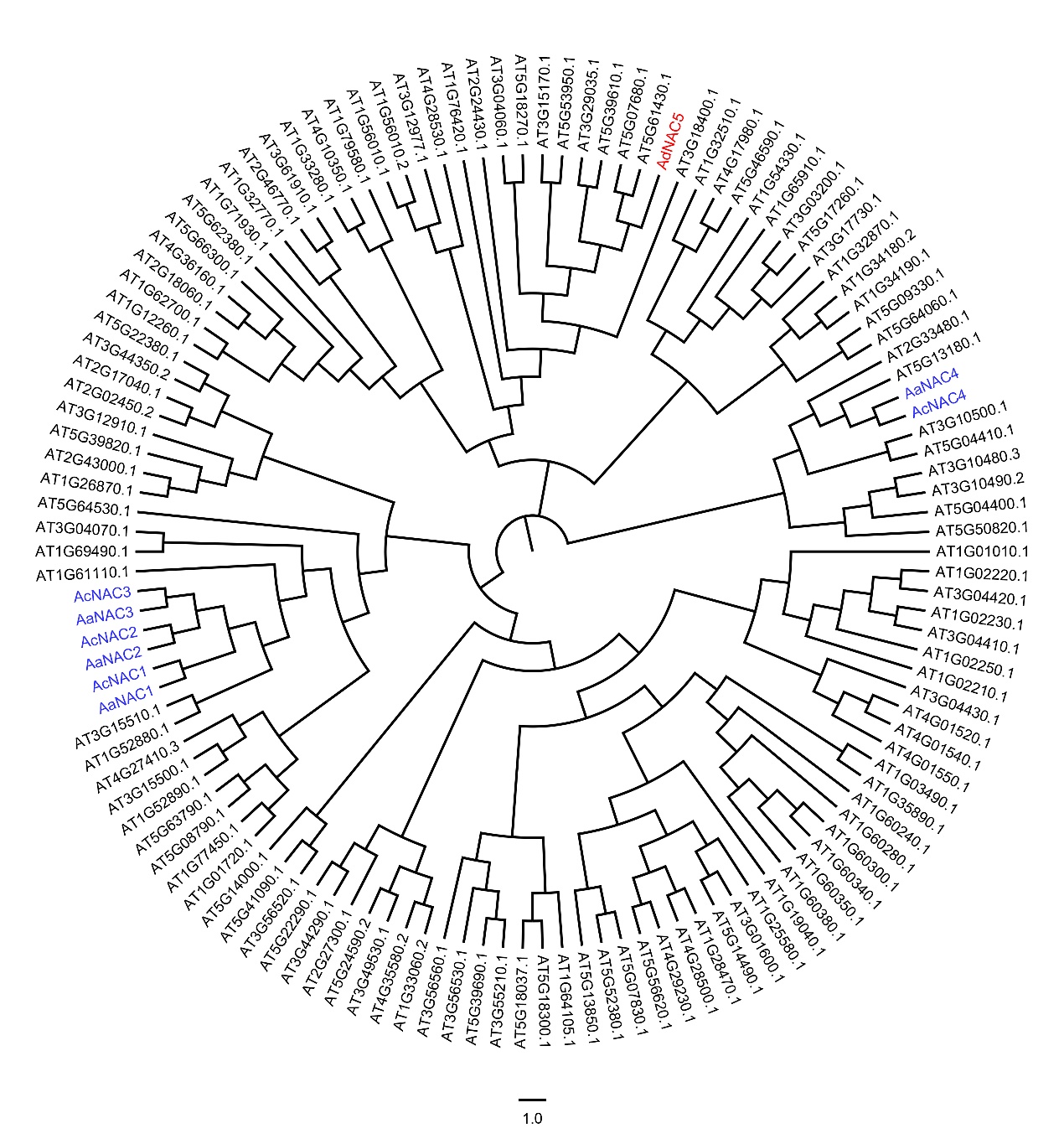
**

**Additional file 2 Fig. S5.** Phylogenetic tree analysis of *AdNAC5* with kiwifruit (*A. chinensis* and *A. arguta*) reported *NACs* (*AaNAC1*, KF319046; *AaNAC2*, KF319047; *AaNAC3*, KF319048; *AaNAC4*, KF319049; *AcNAC1*, KF319050; *AcNAC2*, KF319051; *AcNAC3*, KF319052; *AcNAC4*, KF319053) and *Arabidopsis thaliana*. The deduced amino acid sequences of *Arabidopsis* *thaliana* *NACs* were obtained from TAIR.The phylogenetic trees were constructed using FigTree (v1.4.2).
