# Supplementary material for: Transcriptome co-expression network analysis identifies key genes and regulators of ripening kiwifruit ester biosynthesis
Source: BMC Plant Biol. 2020 Mar 6;20:103. doi: 10.1186/s12870-020-2314-9 (PMC7059668; doi:10.1186/s12870-020-2314-9)
Supplement: Supplementary file 3 — Additional file 3:Table S1. Aroma biosynthesis structural genes. Table S2. Primers for real-time PCR. Table S3. Sequences (5′ to 3′) for promoter isolation. Table S4. Primers for vector construction for dual-luciferase assays. [file 12870_2020_2314_MOESM3_ESM.docx]

**Additional file 1 Table S1.** Aroma biosynthesis structural genes

| Gene | Genome relevant (>95%) | Reference | Full name |
| --- | --- | --- | --- |
| *AdALDH1* | *Acc12304.1* |  | Aldehyde dehydrogenase |
| *AdALDH2* | *Acc11529.1* |  | Aldehyde dehydrogenase |
| *AdADH3* | *Acc21782.1* |  | Alcohol dehydrogenase |
| *AdAT17* | *Acc22635.1* | Souleyre *et al*., 2014 | Alcohol acyl transferase |
| *AdFAD1* | *Acc10173.1* |  | Fatty acid desaturase |
| *AdFAD2* | *Acc18482.1* |  | Fatty acid desaturase |

**Additional file 1 Table S2.** Primers for real-time PCR

| **Gene** | **Forward (5’ to 3’)** | **Reverse (5’ to 3’)** |
| --- | --- | --- |
| ***AdALDH2*** | TGTCACGGTCAACAATCTGGAA | CTCTCCCCAACTCCTCCAAATG |
| ***AdAT17*** | GGCCATAATGACCGGAGAGTATT | TCAGACACAATATAGTTCCCCGC |
| ***AdFAD1*** | TCTCGACAATGCCACATTACCA | ACACCTTTGCTCTGTTCACTCT |
| ***AdActin*** | TGCATGAGCGATCAAGTTTCAAG | TGTCCCATGTCTGGTTGATGACT |

**Additional file 1 Table S3.** Sequences (5’ to 3’) for promoter isolation

| Gene | Promoter sequence | Length (bp) |
| --- | --- | --- |
| *AdAT17* | CAACGGAAATAAGCCGTTCGAGACTCCCATGACCAATTAATGAGGGAAAATATAAAAATCCATTTGATTATACTTACTTGTTTTTGGCCTATTTGGTTTAAGTTTTTCAAATAGCCAACTGATATTTTAGTTTGGTTTAATTTTCTTATTAGGTTTGGTATATATTAATTGTCAACAAATTATCCCGAAGCATTTCCATACACTTTGGGAAAAGAGTGATGGAGAAAGGGAGAAGAGAGATGATAGTGAGGAGATACAAATACGAAGCTGGTGTCAAGTAGGGGAGAGAAGATTGATAGTGGGTGTTGAGAAAAGGGGATTCGGTTAAAGATGTGTTAATTTAATGGATTAAAAAGGCGTAGACAAATAAATTAAAGATATTAAATTTTTTAAACGGGTGTAAAGAGAATGTGAAGAACATAGCCCGTAAAGTAGCTCACTTGCACCTCACAATACGAAAGTATAAAGGTTTGACTGTGCCAGTTTAAAGTTAAGGGAGCAAAGTAAAATTGAGTGCAAAGTTCAGAGGTGCAAAGTATAATTTATGAAAAAAAACTCCCCTATAAATACTTTCCAAAATTTTGATACATGGCATCTCTGCTGTTCTCCTCAGATATTTTATCAAATAGTTTCCTTAACT | **640** |
| *AdFAD1* | GACCATAGAAATTCGGAGTTTCAACAAAATGGGGCGGATAAGCAAAAAGGATCTGCATAGTCTCCCCAAGACTGCACCCTTGGATAGCTCAGTCGCGGGTAAAAATTCTGACTTTATGTTTAATGTATCGTCAATTTGAATCCTTTTACAATTTCTAGATTTTTATCTGACTATCAACTTCAAACACCATTTAAAAACACCATTTAAAGGGATTAGCATTTTTCATATTAAAAAGCTACTATTGTTCAGAAAGGGATAGAAAATTGTTTCTCATTTATCCATGACTCTTCCACTCTTGGAAAGGCCAGTCTGTTTAATGGCATCACCAATTTGAGACCTTTTATGACTTATATTTTTGGACCGTTAATTTCAGACATCTTATTAGTCTCTCGCAAATCTAATTGGACTAGATGAGAATACATGTTATAAAAAAAAGTTGTCCAAGACGCTCAATAGTTTAGTGTTTTTTATTTTATTTTTTAGGAGTCAAAAGAACACTTCTTTGGTCAGTTTTTAGAATTTTTTTTAGAGAAATTTTTATAAAAAGAGCTTATTTTTAAGAAAACAGAAAAAAAATTACTTAAATTCTTTTGTTTTTTAAAAGAAATGTATAAAAAAATTACCATATTATTTTTTTAAAATAATTAAAAAAATATTCTGAAAACAGCAAAGCCTTGTATGTCGTGGCAAGTGCGTATCTTCCGCATTGATTAATGTAAGTTCGTGGTTATCTTGGGATTTGACTCAGTTTTGGGTGGTAAGATTTTATTATTCAGAATTTTTTAAAGGATAATTGATTAATAACTTAATATAAAGATAACTTAAAAGATGCTTGAAACTAAGACTCAAACAGACGGGACTGTTAAAACTAGCCGGACTTTGCGTGGTGTCCAGTCCAAGCCTAATTGGCAATAGGCTTCGGACCAGACACATACTCGAACAGTCCAACCAGACCAACCCACGCCTATTCTGGGCGGGATCTCCGGGCCGGACCCACTGTGCAACCCTAGCGGTTAGGACCAACCCACGCCTATTCTGGGCGGGATCTCCGGGCCGGACCCACTGTGCAACCCTAGCGGTGCCTAACCGCGCTCAGCTCAGATTTTGGATTATTTTTTCTTATATAGCTGATCATTAAATGAAATGCTATCAAAGCTGACAAGGTGACCGAGATCTGATATAAGAATTACTTATAGTACACAAGTGATGTATAGTTTCTTTTTAGAATTTTATTTTATTTTATTTAAGAAAGATCATCATGATAATATGATATCAGTCCTATCAAGTATCAAGGCTTGGGAGAGAAGGAGAAGCACGAAAGAGGCCTGGGACTTGTAATGAGGGTGCCGTGCCTTTAAATGCTCCAACCCCTGGGTCAACCTATAAAGCCGTGTGTTTATTTCGCAAATAACTGAATAAGAAAGTTAGGTGGTGGTGAGAGAAACACGATGTTTTTTGCATTTTTAGTGTATTGTGTTTGATGTTAATTTTGACTACGTGCTGCTAGAAATGTAATAAAAAAATTATTTTTTGTCGTGTCTTGTCAAAAAAGGAAAGTGTGTTCAGATAGAAATTATATATAATATCGCGTGTGAATAATGACATACTAACATGTGTTTGGTTAATTTTTTTTCTCTCTTCTAGATGAGCTTATTTCTTACACATTCCATTTGAATATAGCATGCGCTAGAAGCTCCAATTTTTTATTTTTTTTTTGAGTTAGATACATCCATCTATATGGATTGTGCTCACTGCTATAACCATTTTTTCAGGCCACTGAACA | **1783** |

**Additional file 1 Table S4.** Primers for vector construction for dual-luciferase assays

| **Gene** | **Forward (5’ to 3’)** | **Reverse (5’ to 3’)** |
| --- | --- | --- |
| *AdAT17* | TCCACTAGTTCTAGAGCGGCCGCCAACGGAAATAAGCCGTTCGAGA | TGTTTTTGGCGTCTTCCATGGAGTTAAGGAAACTATTTGAT |
| *AdFAD1* | TCCACTAGTTCTAGAGCGGCCGCGACCATAGAAATTCGGAGTTTC | TGTTTTTGGCGTCTTCCATGGTGTTCAGTGGCCTGAAAAAATGG |
